# SM-GAT: a safety-aware multi-task graph attention network for multi-target anti-diabetic lead discovery from natural products

**DOI:** 10.3389/fmolb.2026.1854162

**Published:** 2026-07-08

**Authors:** Jianxin Zhang, Hongyi Liu, Shengnan Guo

**Affiliations:** 1 The Second Qilu Hospital of Shandong University, Jinan, China; 2 The First Clinical School of Medicine, Yunnan University of Traditional Chinese Medicine, Kunming, China

**Keywords:** anti-diabetic activities, graph attention network, multi-task learning, natural products, virtual screeening

## Abstract

Traditional Chinese Medicine constitutes a chemically diverse and pharmacologically rich reservoir of bioactive compounds, often exhibiting multi-target pharmacological properties that are potentially valuable for complex metabolic disorders such as type 2 diabetes mellitus (T2DM). However, systematic prioritization of active and safe constituents remains challenging, as therapeutic efficacy must be optimized concurrently with toxicity risk. Here, we present a safety-aware Multi-Task Graph Attention Network (SM-GAT) framework that jointly models anti-diabetic efficacy and toxicity liabilities of TCM-derived compounds within a unified architecture. Four tasks are simultaneously optimized: inhibition of dipeptidyl peptidase-4 (DPP4), inhibition of α-glucosidase, acute oral toxicity, and clinically relevant toxicity. By enabling shared molecular representation learning across heterogeneous yet biologically related endpoints, SM-GAT facilitates knowledge transfer between efficacy and safety domains. Across all prediction tasks, SM-GAT achieved competitive or superior performance compared with single-task graph neural networks and other baseline models, achieving ROC-AUC values up to 0.892 for α-glucosidase inhibition. Notably, multi-task learning yields pronounced improvements in data-limited settings, highlighting effective cross-task regularization. Large-scale virtual screening of the TCMBank library demonstrates practical applicability, enabling efficient prioritization of structurally diverse candidates with favorable predicted efficacy–safety balance. Several structurally diverse lead compounds, including ellagic acid derivatives, are identified with favorable predicted efficacy–safety balance. Furthermore, atom-level attention analysis highlighted chemically interpretable substructures associated with predicted efficacy and toxicity-related molecular representations. Collectively, this study establishes an interpretable multi-objective framework for safety-aware lead discovery, providing a computational framework for integrating traditional botanical resources into anti-diabetic lead discovery.

## 1 Introduction

Type 2 diabetes mellitus (T2DM) is a chronic metabolic disorder characterized by persistent hyperglycemia and progressive impairment of glucose regulation (Zheng et al., 2018; Lascar et al., 2018). Owing to its multifactorial etiology—encompassing dysregulated insulin secretion, impaired incretin signaling, and altered carbohydrate metabolism—effective long-term management frequently requires simultaneous modulation of multiple biological pathways (Walker et al., 2023; Mizukami and Kudoh, 2022; Suzuki et al., 2024). Among validated therapeutic mechanisms, dipeptidyl peptidase-4 (DPP4) and α-glucosidase play complementary roles in glycemic control (Min et al., 2018). DPP4 regulates incretin hormone degradation and thereby influences insulin secretion, whereas α-glucosidase mediates intestinal carbohydrate hydrolysis and postprandial glucose absorption. Although inhibitors of these enzymes are widely used in clinical practice, synthetic agents are often associated with gastrointestinal intolerance, off-target effects, and potential long-term safety concerns (Mulvihill, 2018; Gao et al., 2018). The identification of multi-target agents with improved safety profiles therefore remains an unmet clinical need.

Safety considerations are particularly critical in T2DM, as pharmacological interventions are typically administered chronically. A significant proportion of drug candidates fail in late-stage development due to unforeseen toxicities, including hepatotoxicity, nephrotoxicity, and systemic adverse reactions (Waring et al., 2015; Kola and Landis, 2004). Early-stage integration of toxicity assessment into lead discovery is thus essential to reduce attrition and improve translational success. While experimental toxicological evaluation remains indispensable, it is resource-intensive and not readily scalable to large chemical libraries. Computational toxicity prediction has consequently emerged as a key strategy for preclinical risk mitigation, enabling rapid identification and exclusion of high-liability compounds (Bueso-Bordils et al., 2024; Zhang et al., 2025).

Traditional Chinese Medicine (TCM) represents a historically validated yet underexplored source of chemically diverse small molecules. Unlike the conventional “one drug–one target” paradigm, TCM-derived compounds often exhibit polypharmacological properties arising from complex structural scaffolds and evolutionary selection within botanical systems (Zhai et al., 2025; Li and Zhang, 2013). This intrinsic multi-target potential renders TCM particularly attractive for complex diseases such as T2DM. The recent digitization of TCM resources, including comprehensive repositories such as TCMBank, has created unprecedented opportunities for large-scale computational screening (Lv et al., 2023a; Lv et al., 2023b). Nevertheless, the structural heterogeneity and compositional complexity of TCM compounds pose substantial challenges for systematic prioritization, especially when efficacy and safety must be optimized concurrently (Wang et al., 2024; Li et al., 2025; Fu, 2021).

Recent advances in graph-based deep learning have substantially improved molecular property prediction by directly operating on molecular graph representations (Shen et al., 2023; Zhao et al., 2024). In graph attention networks (GATs), atoms and bonds are encoded as nodes and edges, respectively, while attention mechanisms dynamically weight local chemical environments during representation learning. This architecture preserves molecular topology and captures context-dependent substructural features critical for both activity and toxicity prediction. However, most existing studies treat efficacy and safety endpoints independently, thereby overlooking shared molecular determinants and failing to leverage potential cross-task information (Zhao et al., 2020; Liu et al., 2022).

To address these limitations, we propose a safety-aware multi-task learning framework based on Graph Attention Networks for TCM-derived anti-diabetic lead discovery. By jointly modeling DPP4 inhibition, α-glucosidase inhibition, acute oral toxicity, and clinical toxicity within a unified architecture, the proposed SM-GAT framework enables shared representation learning across partially overlapping datasets. This design facilitates cross-domain knowledge transfer between efficacy and toxicity endpoints while improving robustness under experimental variability. Through comprehensive benchmarking and large-scale virtual screening, we demonstrate that multi-objective optimization within a graph attention framework provides an effective and interpretable strategy for prioritizing TCM-derived compounds with balanced therapeutic indices.

## 2 Materials and methods

### 2.1 Dataset collection and task definition

#### 2.1.1 Bioactivity and toxicity data

To enable safety-aware multi-objective modeling, we constructed a heterogeneous multi-task dataset integrating anti-diabetic efficacy and chemical toxicity endpoints. Bioactivity records were collected from ChEMBL (Mendez et al., 2019), focusing on two clinically validated therapeutic targets for type 2 diabetes mellitus: human dipeptidyl peptidase-4 (DPP4) and α-glucosidase. Experimentally measured IC_50_ values were extracted and converted into binary activity labels using three commonly adopted potency thresholds (100 nM, 500 nM, and 1,000 nM), corresponding to high-, medium-, and low-stringency definitions of activity. Evaluating multiple thresholds enabled systematic assessment of model robustness under varying levels of experimental uncertainty and label noise. Toxicity annotations were obtained from the TOXRIC (Wu et al., 2023) database and included two complementary safety endpoints: acute oral toxicity (LD_50_-based OralTox) and clinically relevant toxicity (ClinicalTox).

#### 2.1.2 Molecular standardization

All molecular structures were standardized using RDKit, including salt removal, charge neutralization, and valence correction, to ensure chemical consistency across heterogeneous data sources. Canonical SMILES representations were used throughout the study. Because efficacy and toxicity annotations were derived from independent repositories, task labels were incomplete and unevenly distributed across compounds. To accommodate this heterogeneity, a task-specific masking strategy was adopted: missing labels were assigned a mask value of −1 and excluded from loss computation and gradient propagation during training. The final curated dataset comprised 16,669 unique compounds, forming a sparse yet structurally aligned benchmark suitable for multi-task representation learning. Before splitting, exact duplicate canonical SMILES were removed during curation. The train/validation/test split was generated by stratified random splitting on the curated unique-compound set based on available task labels while ignoring masked entries.

### 2.2 Molecular graph construction and feature encoding

Each standardized molecule was represented as an undirected molecular graph, where nodes correspond to atoms and edges correspond to covalent bonds. Molecular graphs were generated using RDKit, and self-loops were added to preserve central atomic identity during message passing and neighborhood aggregation. Each atom was initialized with a compact feature vector designed to capture essential chemical properties while minimizing feature leakage. The encoded atom-level features included: (i) atomic number normalized by a factor of 10, (ii) atomic degree defined as the number of bonded neighbors, (iii) formal charge assigned by RDKit, (iv) hybridization state encoded using one-hot categorical features (SP, SP2, SP3, SP3D, SP3D2), and (v) aromaticity represented as a binary indicator. This minimalist feature design encourages the graph attention mechanism to learn task-relevant representations directly from molecular topology and local chemical context. A summary of task definitions, dataset composition, and atomic features is provided in Supplementary Table S1.

### 2.3 SM-GAT architecture and multi-task learning framework

The proposed framework employs a multi-task graph attention network to dynamically weight atomic neighborhood contributions across multiple prediction tasks (Velickovic et al., 2017). Let $X\in R^{N\times F}$ denote the atom-feature matrix and $A\in R^{N\times N}$ the adjacency matrix of a molecular graph with N atoms and F input features. The hidden representation after the first GAT layer is $H^{\left(1\right)}\in R^{N\times d_{h}}$ , and the final graph embedding is obtained by mean pooling as $h_{g}\in R^{d_{h}}$ . Each task head outputs a scalar prediction $y_{t}\in R$ . For a pair of connected atoms *i* and *j*, the unnormalized attention coefficient $e_{ij}$ is computed as:

$$e_{ij}=\text{LeakyReLU}\left(a^{⊤}\left[Wh_{i}∥Wh_{j}\right]\right)$$ (1)

where $h_{i}$ and $h_{j}$ denote node feature embeddings, W is a learnable linear transformation, a is the attention weight vector, and ∥ denotes concatenation. The resulting graph-level embedding $h_{g}$ is a compact summary of the entire molecular graph. Unlike architectures with an explicit learnable super node, mean pooling aggregates node information after message passing. This strategy is permutation invariant, computationally efficient, and applicable to molecules of varying sizes. Because all node embeddings contribute equally, important local substructures may be partially diluted. The graph attention mechanism already assigns different weights to neighboring atoms during message passing, partially mitigating this limitation before graph-level aggregation. The attention coefficients are normalized across the neighborhood $N_{i}$ using a softmax function:

$$\alpha_{ij}=\frac{\exp\left(e_{ij}\right)}{\sum_{k\in N_{i}}\exp\left(e_{ik}\right)}$$ (2)

To enhance representational diversity and training stability, the first GAT layer employed eight independent attention heads, followed by a single-head graph attention layer for feature aggregation. The network consists of stacked GAT layers followed by a global mean pooling operation to aggregate node-level embeddings into a fixed-length graph representation. The resulting 128-dimensional molecular embeddings were subsequently passed to task-specific output heads for binary classification.

To address label sparsity and severe class imbalance, a masked weighted binary cross-entropy loss was adopted. A binary mask $M_{n,t}$ was defined such that $M_{n,t}=1$ if a valid label exists for sample *n* on task *t*, and $M_{n,t}=0$ otherwise. Task-specific positive class weights were computed as:

$$w_{t}=\min\left(\frac{N_{neg,t}}{N_{pos,t}},15.0\right)$$ (3)

where $N_{\text{pos},t}$ and $N_{\text{neg},t}$ denote the numbers of positive and negative samples for task *t*, respectively. To avoid unstable optimization under extreme imbalance, the positive-class weight was capped at 15.0. The total loss function was defined as:

$$L_{\text{total}}=\frac{1}{\sum_{n,t}M_{n,t}}\sum_{n,t}M_{n,t}\cdot L_{\text{BCE}}\left(y_{n,t},{\hat{y}}_{n,t}\right)$$ (4)

ensuring that only observed labels contribute to model optimization while maintaining robustness against extreme class imbalance.

### 2.4 Training protocol and experimental setup

All experiments were conducted on a high-performance Linux cluster running Ubuntu 22.04LTS, equipped with four NVIDIA RTX 4090 GPUs (24 GB VRAM each). The model was implemented using PyTorch 2.1 and the Deep Graph Library (DGL) 2.0. The dataset was partitioned into training, validation, and test sets using an 8:1:1 split. Class imbalance was instead addressed through the task-specific weighted binary cross-entropy loss, which increases the contribution of rare positive samples whenever they are observed during training. Model optimization was carried out using the AdamW optimizer with an initial learning rate of $1\times 10^{-3}$ and a weight decay of $1\times 10^{-4}$ . Gradient accumulation over eight steps was applied to stabilize training under limited GPU memory. A ReduceLROnPlateau scheduler was used to adaptively adjust the learning rate based on validation performance. Hyperparameters, including hidden dimensions, number of attention heads, and dropout rates, were optimized *via* grid search under each activity threshold setting (100 nM, 500 nM, and 1,000 nM). The final configurations are summarized in Supplementary Table S2.

### 2.5 Model evaluation and ablation analysis

Model performance was evaluated using the area under the receiver operating characteristic curve (ROC-AUC) and the area under the precision–recall curve (PR-AUC), which are particularly suitable for highly imbalanced classification tasks. To quantify the contribution of different architectural components, we compared SM-GAT against several representative ablation and baseline models under identical data splits and evaluation metrics, including: ST-GAT (single-task learning without multi-task parameter sharing), MT-GCN (multi-task graph learning without attention mechanism), MT-MLP (multi-task learning without graph-based encoder), and Chemprop (Swanson et al., 2024; Yang et al., 2019) (a widely used multi-task message-passing neural network baseline for molecular property prediction). These comparisons enabled assessment of the contributions of multi-task learning, graph representation learning, and attention-based neighborhood weighting within the proposed architecture. All baseline models were trained under the same data split, optimization protocol, and evaluation metrics as SM-GAT to ensure fair comparison. Hyperparameters were tuned within the same search space where applicable. Performance differences between SM-GAT and single-task baselines were used to assess the benefit of shared representation learning, particularly for data-scarce tasks.

### 2.6 Interpretability and virtual screening application

Model reliability and practical applicability were further assessed through interpretability analysis and large-scale virtual screening. The applicability domain (AD) of the model was defined using ECFP4-based Tanimoto similarity, where compounds with a maximum similarity below 0.4 to the training set were considered outside the reliable prediction space.

For structural interpretability, attention coefficients from the final GAT layer were extracted and projected onto two-dimensional molecular depictions, enabling identification of substructures that contribute most strongly to predicted activity or toxicity. This facilitates interpretation of chemically relevant substructures associated with predicted activity or toxicity.

Finally, the optimized SM-GAT model was applied to screen 29,907 natural compounds from the TCMBank database. Compounds were prioritized as candidate leads if they exhibited high predicted activity probabilities (P > 0.8) against both anti-diabetic targets while simultaneously maintaining negative toxicity predictions across safety-related tasks. To prioritize compounds for simultaneous efficacy and safety, we defined a composite ranking score. Specifically, for each compound, the efficacy score was calculated as the average of the predicted DPP4 and α-glucosidase inhibition probabilities. The toxicity penalty was defined as the maximum of the predicted oral-toxicity and clinical-toxicity probabilities. The final composite ranking score was explicitly defined as:

$$S=\frac{\left(P_{\text{DPP}4}+P_{\alpha ‐\text{glu}}\right)}{2}\times \left(1-\max\left(T_{\text{oral}},T_{\text{clinical}}\right)\right)$$ (5)

This multiplicative penalty form ensures that high predicted efficacy cannot compensate for severe predicted toxicity, which is desirable for early-stage anti-diabetic lead prioritization. Compounds with high predicted efficacy and low predicted toxicity were prioritized as top-ranked candidates under the composite score, followed by complementary inspection of the full multi-task prediction profile.

## 3 Results and discussion

### 3.1 Overall predictive performance across efficacy and toxicity tasks

The proposed SM-GAT framework integrates multi-task learning with graph attention mechanisms to jointly model heterogeneous efficacy and safety endpoints within a unified molecular representation space. By enabling parameter sharing across partially labeled datasets and adaptively weighting local chemical environments, the architecture is designed to capture shared structural determinants underlying both pharmacological activity and toxicity risk. To systematically evaluate predictive capability, we assessed model performance on a curated dataset of 16,669 compounds spanning two anti-diabetic efficacy targets (DPP4 and α-glucosidase) and two toxicity endpoints (acute oral toxicity and clinical toxicity).

Using 500 nM as the primary bioactivity threshold, SM-GAT achieved consistent and well-balanced discriminative performance across all four tasks (Figure 2A). ROC-AUC values reached 0.762 for DPP4 inhibition and 0.892 for α-glucosidase inhibition. Notably, α-glucosidase represents the smallest efficacy dataset in terms of labeled samples, yet yielded the highest ROC-AUC, suggesting that shared multi-task representation learning may contribute to improved performance. For toxicity prediction, ROC-AUC values of 0.699 (oral toxicity) and 0.821 (clinical toxicity) were achieved despite pronounced class imbalance. The ability to maintain strong discrimination under skewed label distributions indicates that the masked weighted loss formulation effectively mitigates bias toward majority classes. Because toxicity endpoints often exhibit extremely low positive prevalence, we further evaluated model behavior using precision–recall analysis (Figure 2B). SM-GAT maintained stable precision across a broad recall spectrum, particularly for clinical toxicity, demonstrating robustness in low-prevalence regimes. Under a fixed 90% specificity constraint (Figure 2C), SM-GAT retained 92% sensitivity for α-glucosidase and 62% for clinical toxicity, indicating that high-stringency early screening can be achieved without excessive loss of true positives.

**FIGURE 1:**
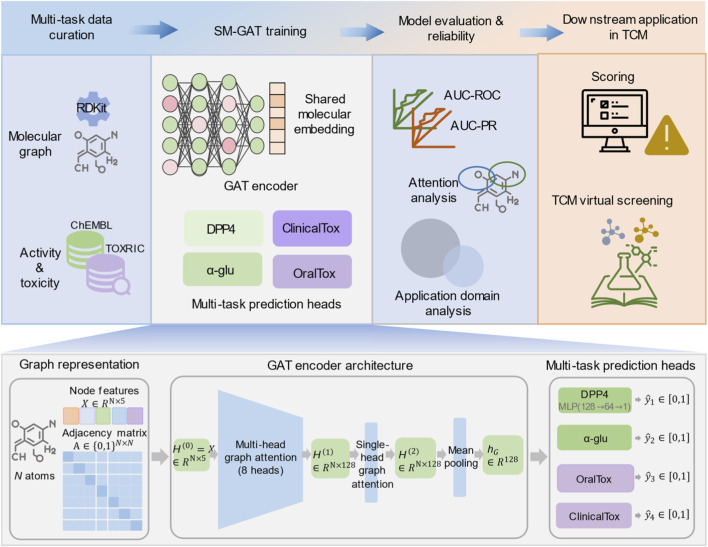
Overview of the SM-GAT framework for safety-aware multi-objective anti-diabetic lead discovery. The workflow comprises four sequential stages: (1) Multi-task data curation, integrating anti-diabetic efficacy data (DPP4 and α-glucosidase inhibition) with toxicity annotations (acute oral and clinical toxicity), followed by molecular graph construction. (2) SM-GAT training, where a shared graph attention encoder learns shared molecular embeddings optimized jointly by multiple task-specific prediction heads. (3) Model evaluation and reliability analysis, including ROC-AUC and PR-AUC assessment, attention-based interpretability, and applicability domain analysis. (4) Downstream virtual screening, where the trained model is applied to large-scale TCM compound libraries to prioritize candidates with balanced efficacy–toxicity profiles. The lower panel illustrates the detailed SM-GAT architecture. Each molecule is represented as a graph with atom-feature matrices and adjacency matrices as inputs. The shared encoder comprises a multi-head graph attention module (8 heads) followed by a single-head graph attention module, producing node-level representations that are aggregated through mean pooling into a 128-dimensional shared molecular embedding. The resulting embedding is subsequently processed by task-specific multilayer perceptron heads for DPP4 inhibition, α-glucosidase inhibition, OralTox, and ClinicalTox prediction.

**FIGURE 2:**
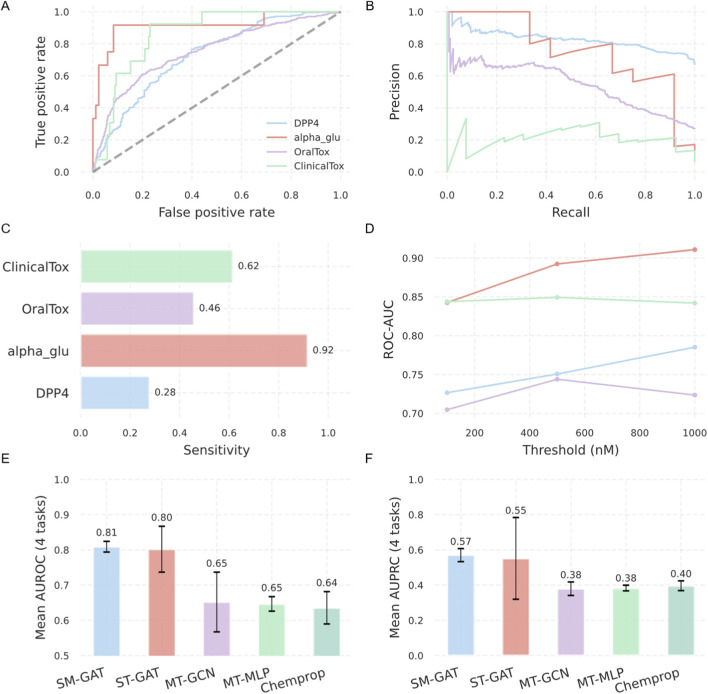
Predictive performance and virtual screening evaluation of SM-GAT. **(A)** ROC curves for four prediction tasks under internal validation. **(B)** Precision–recall curves highlighting performance under severe class imbalance. **(C)** Sensitivity evaluated at 90% specificity, reflecting high-stringency early screening conditions. **(D)** Stability of ROC-AUC across bioactivity thresholds (100, 500, and 1,000 nM). Mean AUROC. **(E)** and mean AUPRC. **(F)** comparison between SM-GAT, single-task GAT (ST-GAT), and other multi-task deep learning baselines at 500 nM across five independent runs. Also see Supplementary Figure S1 for results on the cleaned test set (filtered at Tanimoto similarity ≥0.9).

We next evaluated model robustness across different activity definitions by varying the bioactivity threshold from 100 nM to 1,000 nM (Figure 2D). Predictive performance for DPP4 and α-glucosidase gradually improved as the threshold became less stringent, while ClinicalTox remained relatively stable across thresholds. OralTox exhibited only minor fluctuations, suggesting that the learned representations are not strongly dependent on a single activity cutoff.

To further assess the effectiveness of the proposed architecture, SM-GAT was compared with several baseline models, including ST-GAT, MT-GCN, MT-MLP, and Chemprop (Figures 2E,F). SM-GAT achieved competitive overall performance, reaching a mean AUROC of 0.81 and a mean AUPRC of 0.57 across the four tasks. Although ST-GAT achieved comparable AUROC values, its AUPRC remained slightly lower, while MT-GCN, MT-MLP, and Chemprop showed substantially weaker overall performance. Compared with independently trained single-task models, the multi-task framework showed consistent gains particularly for toxicity-related tasks, suggesting potential knowledge transfer from efficacy-rich tasks to data-scarce safety tasks. MT-GCN underperformed relative to SM-GAT, suggesting that attention-based neighborhood weighting contributes to improved representation quality beyond standard graph convolution. MT-MLP exhibited substantially weaker performance, highlighting the importance of topology-aware graph representations for capturing chemically relevant local context. These results demonstrate that shared representation learning within SM-GAT improves generalization across heterogeneous biochemical and toxicological tasks, establishing a multi-endpoint framework for simultaneous efficacy–toxicity screening in anti-diabetic lead discovery.

### 3.2 Benefits of multi-task learning under heterogeneous data regimes

**FIGURE 3:**
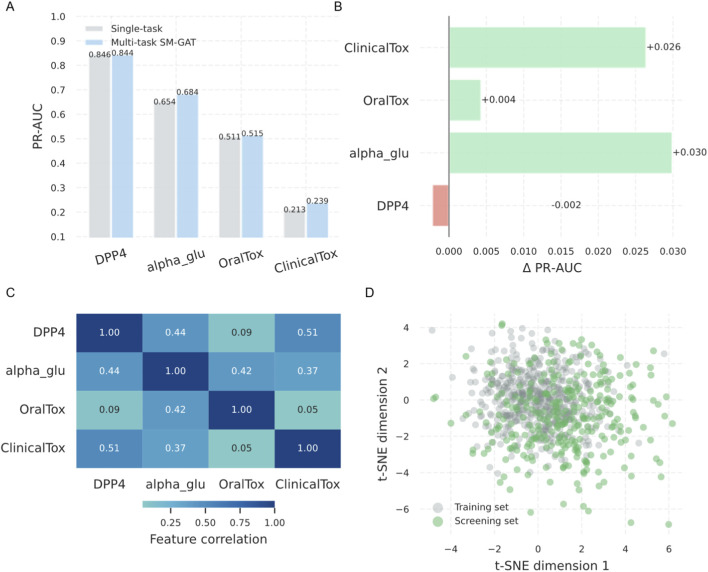
Multi-task learning benefits and applicability domain analysis. **(A)** PR-AUC comparison between SM-GAT and corresponding single-task GAT models at 500 nM. **(B)** Task-wise PR-AUC improvements attributable to multi-task learning. **(C)** Inter-task feature correlation heatmap derived from shared latent representations, revealing moderate structural dependencies between efficacy and toxicity tasks. **(D)** Applicability domain assessment based on t-SNE projection, illustrating chemical space overlap between the training dataset and the TCMBank screening library.

To quantify the contribution of multi-task learning under partially labeled and heterogeneous data conditions, SM-GAT was compared with independently trained single-task GAT models using identical architectures and optimization protocols. As shown in Figure 3A and Supplementary Table S5, joint learning improved predictive performance across most endpoints under PR-AUC evaluation. The largest improvement was observed for α-glucosidase inhibition, where multi-task training yielded an absolute PR-AUC increase of +0.030 relative to the single-task baseline (Figure 3B). ClinicalTox prediction also showed a notable improvement (+0.026), whereas OralTox exhibited a smaller positive gain (+0.004). In contrast, DPP4 performance remained essentially unchanged (−0.002), suggesting that the data-rich DPP4 task already possessed sufficiently strong task-specific supervision and therefore benefited less from auxiliary-task transfer. Given the limited annotation size for α-glucosidase and ClinicalTox, these gains are consistent with beneficial cross-task information sharing through shared molecular representations.

To explore the structural basis of this transfer, we analyzed inter-task feature correlations derived from shared latent representations (Figure 3C). Moderate correlations (0.42–0.51) were observed between efficacy and toxicity tasks, indicating partially shared structural determinants. Such intermediate correlation levels may facilitate effective shared representation learning: sufficient overlap to enable shared learning, yet distinct enough to preserve task-specific specialization. This balance likely mitigates overfitting while improving generalization on sparse endpoints.

Beyond performance gains, multi-task learning also strengthened generalization reliability. Applicability domain analysis was conducted by projecting both training compounds and the TCMBank library into a two-dimensional chemical space using t-SNE (Figure 3D). The majority of screening compounds were embedded within the structural manifold defined by the training data, suggesting substantial overlap between the screening library and the training chemical space, although t-SNE visualization alone does not constitute a formal applicability-domain guarantee. These findings demonstrate that SM-GAT leverages shared yet task-aware representations to improve performance under heterogeneous labeling, particularly for data-limited endpoints, while enhancing the robustness and reliability of large-scale virtual screening.

### 3.3 Training stability and interpretability of SM-GAT

**FIGURE 4:**
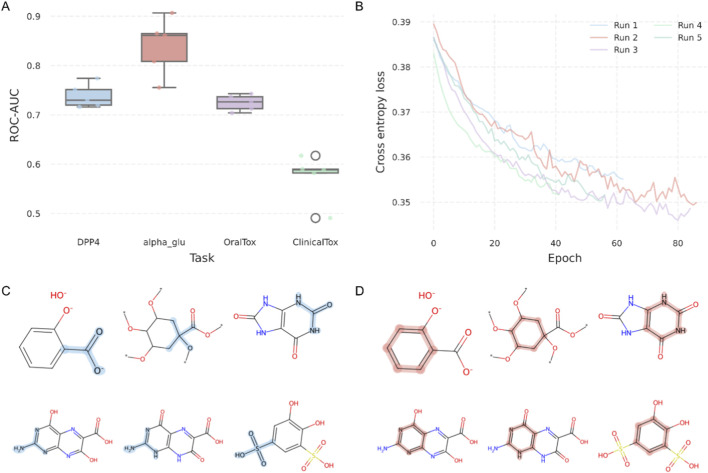
Optimization stability and attention-based interpretability of SM-GAT. **(A)** ROC-AUC distributions across five independent training runs, demonstrating reproducible predictive performance. **(B)** Convergence trajectories of masked binary cross-entropy loss across runs, indicating stable and well-conditioned multitask optimization. **(C)** Atomic-level attention visualization for representative compounds in the DPP4 task, highlighting substructures contributing most strongly to predicted efficacy. **(D)** Attention maps under the multitask setting, showing more distributed weight allocation across molecular scaffolds to jointly capture efficacy- and toxicity-related structural signals.

To assess robustness under stochastic optimization, five independent training runs were performed using different random initializations and data splits. As summarized in Figure 4A, SM-GAT shows generally stable performance across independent runs, although the α-glucosidase task exhibits larger variance due to its substantially smaller labeled sample size. The narrow interquartile ranges and minimal deviation across runs suggest that the shared representation learning is not sensitive to initialization noise or partitioning artifacts, supporting training robustness under heterogeneous labeling. Training dynamics further confirmed optimization stability. As shown in Figure 4B, masked binary cross-entropy loss decreased smoothly and consistently across all runs, with convergence typically achieved within 40–60 epochs. No obvious oscillatory behavior or training divergence was observed. The similarity of convergence trajectories across runs indicates that the multitask objective is well-conditioned and that the shared–task-specific architecture does not exhibit obvious optimization instability during training, a common concern in multi-objective learning settings.

Beyond numerical stability, we investigated model interpretability through atomic-level attention analysis. Attention weights from the final graph attention layer were projected onto representative molecular structures to identify substructures contributing most strongly to predictions. For the DPP4 task, attention was concentrated on heteroaromatic scaffolds, conjugated systems, and adjacent polar functionalities (Figure 4C). These motifs partially overlap with reported structural features associated with DPP4 inhibition involving hydrogen bonding and π–π interactions within the enzyme active site, suggesting that the learned attention patterns capture chemically meaningful structural features (Syam et al., 2021; Bayanati et al., 2024). Under the multitask setting, attention distributions became more spatially distributed across molecular graphs (Figure 4D). Rather than focusing narrowly on a single pharmacophoric core, the model assigned non-negligible weights to multiple functional regions, reflecting the need to simultaneously encode efficacy-related and toxicity-associated structural signals. This redistribution suggests that multitask learning encourages broader contextual encoding, promoting more balanced feature utilization across shared and task-specific structural patterns. These findings demonstrate that SM-GAT achieves stable convergence, reproducible performance, and chemically meaningful interpretability. The combination of optimization robustness and mechanistic transparency strengthens confidence in its deployment for multitask drug discovery and multi-objective lead prioritization.

### 3.4 Knowledge-guided validation using traditional Chinese medicine

**FIGURE 5:**
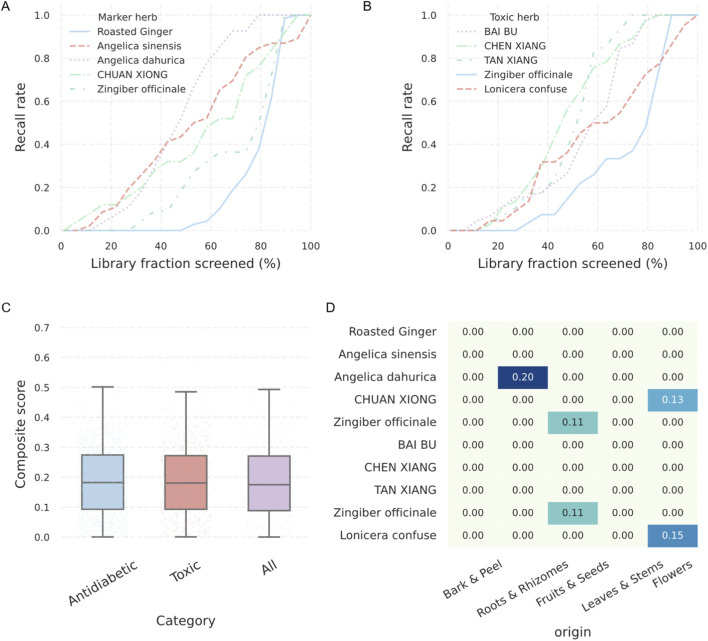
Knowledge-guided external validation of SM-GAT predictions using TCMBank. **(A)** Recall curves for compounds derived from classical anti-diabetic (“Xiao Ke”) marker herbs as a function of library screening depth. **(B)** Recall curves for compounds from toxicity-associated herbs, illustrating delayed enrichment due to predicted safety penalties. **(C)** Composite score distributions across anti-diabetic-associated herbs, toxicity-associated herbs, and the overall compound library. **(D)** Multi-component enrichment analysis across botanical species and plant parts, showing mean composite scores for representative herb–origin combinations.

To further assess the biological plausibility of model predictions beyond supervised benchmark evaluation, we conducted a knowledge-guided validation using empirical associations embedded in the TCMBank resource. Rather than relying on additional experimental annotations, this strategy leverages historically documented relationships between herbs, therapeutic indications, and toxicity awareness as a complementary knowledge-based evaluation framework. We first analyzed recall curves for compounds derived from classical anti-diabetic (“Xiao Ke”) marker herbs as a function of screening depth (Figure 5A). Constituents from well-established anti-diabetic herbs—including *Angelica sinensis* (Wang et al., 2015), *Angelica dahurica* (Zhao et al., 2022), *Ligusticum chuanxiong* (Qi et al., 2024), and *Glycyrrhiza uralensis* (Radix Glycyrrhizae) (Yang et al., 2020)—were preferentially enriched among top-ranked candidates. These herbs exhibited steeper early recall slopes compared with background compounds, indicating that SM-GAT effectively prioritizes chemical constituents aligned with traditional anti-diabetic indications despite no explicit TCM supervision during training.

Conversely, compounds originating from herbs historically associated with toxicity or adverse reactions showed delayed recall enrichment (Figure 5B). Toxic marker herbs such as *Stemona japonica* (Bai Bu) (Wang et al., 2022), *Aquilaria sinensis* (Chen Xiang) (Chan et al., 2024), and *Santalum album* (Tan Xiang) (Burdock and Carabin, 2008) were underrepresented among early high-ranking candidates and became enriched primarily at deeper screening fractions. This pattern suggests that the composite scoring strategy appropriately penalizes predicted toxicity risk, enabling discrimination between efficacy-associated and toxicity-associated chemical signatures. To quantitatively compare biological categories, composite score distributions were analyzed across anti-diabetic herbs, toxicity-associated herbs, and the overall compound set (Figure 5C). Anti-diabetic-associated compounds displayed a right-shifted distribution with higher median scores, whereas toxicity-associated compounds showed comparatively lower composite values. The intermediate positioning of the full library distribution indicates balanced model calibration rather than systematic bias toward a specific class.

Finally, multi-component enrichment was examined across botanical origins and plant parts (Figure 5D). Certain herb–tissue combinations, particularly roots and rhizomes from anti-diabetic-associated species (Ardalani et al., 2021; Ashagrie et al., 2025), demonstrated elevated mean composite scores, reflecting localized accumulation of chemically favorable constituents. Importantly, enrichment was not uniform across plant parts, suggesting that the model captures chemically meaningful heterogeneity consistent with phytochemical distribution patterns. The concordance between SM-GAT predictions and independent TCM knowledge—including therapeutic indication, toxicity awareness, and botanical specificity—provides external validation that the model encodes pharmacologically relevant structural patterns beyond its supervised training labels. This knowledge-aligned generalization supports its applicability in natural product–driven multi-objective drug discovery.

### 3.5 Identification of high-confidence TCM-derived lead candidates

**FIGURE 6:**
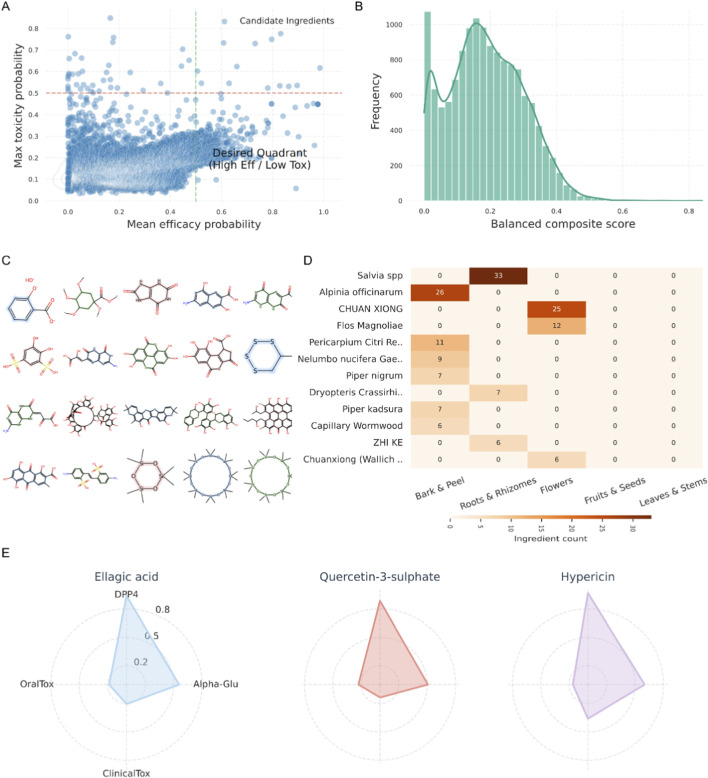
Multi-objective prioritization of multitarget TCM-derived lead candidates. **(A)** Integrated efficacy–toxicity landscape for TCMBank compounds. Each point represents a screened compound plotted by mean predicted DPP4/α-glucosidase inhibition probability (y-axis) *versus* maximum predicted toxicity probability (x-axis). Dashed lines delineate the high-efficacy/low-toxicity prioritization region. **(B)** Distribution of composite balancing scores integrating multi-target efficacy and toxicity penalties, illustrating global ranking characteristics across the screened library. Candidates are ranked by the composite score. **(C)** Chemical structures of representative top-ranked compounds, highlighting structural diversity among prioritized multitarget leads. **(D)** Heatmap summarizing the distribution of prioritized compounds across medicinal plant species and plant tissues, indicating broad botanical coverage. **(E)** Radar plots depicting multi-endpoint prediction profiles of representative leads, including DPP4 inhibition, α-glucosidase inhibition, acute oral toxicity, and clinical toxicity probabilities.

To prioritize high-confidence anti-diabetic leads, the optimized SM-GAT model was deployed to screen the entire TCMBank library. An integrated efficacy–toxicity decision landscape was constructed by projecting each compound onto a two-dimensional space defined by its mean predicted inhibitory probability against DPP4 and α-glucosidase and its maximum predicted toxicity probability across all safety endpoints (Figure 6A). This projection enables simultaneous visualization of therapeutic potential and safety liability at the library scale. Compounds exhibiting high predicted efficacy and low predicted toxicity were prioritized for downstream ranking and analysis. To further refine prioritization within this favorable region, we computed a composite ranking score that quantitatively integrates multi-target efficacy and toxicity penalties into a single ranking metric. The score distribution exhibited a pronounced right-skewed pattern (Figure 6B), indicating that only a limited subset of compounds simultaneously achieved strong predicted inhibition across both targets while maintaining minimal toxicity risk. This distribution pattern supports the stringency of the multi-objective optimization strategy and suggests effective discrimination by the model rather than uniform score inflation. Structural analysis of the highest-ranked compounds revealed substantial chemical diversity (Figure 6C), encompassing polyphenolic frameworks, flavonoid derivatives, phenolic acids, and complex polycyclic scaffolds. The absence of convergence toward a single dominant chemotype suggests that SM-GAT captures generalized structure–activity–toxicity relationships rather than overfitting to narrow structural motifs. Such diversity is particularly advantageous for downstream lead optimization, as it preserves multiple chemically distinct starting points.

We next evaluated the botanical origins of the prioritized leads (Figure 6D). The selected compounds were distributed across diverse medicinal species and plant tissues—including roots, rhizomes, bark, flowers, and fruits—without obvious source-specific enrichment. This result indicates that the prioritization framework does not introduce systematic bias toward particular botanical categories and is broadly applicable across heterogeneous TCM-derived chemical space. To illustrate representative prediction profiles, three top-ranked compounds—ellagic acid (Les et al., 2018), quercetin-3-sulphate (Zheng et al., 2024), and hypericin (Dong et al., 2021)—were examined in detail (Figure 6E). All three compounds demonstrated high predicted inhibitory probabilities against both DPP4 and α-glucosidase while maintaining comparatively low predicted risks for acute oral and clinical toxicity. Among them, ellagic acid displayed consistently elevated multi-target efficacy coupled with one of the lowest toxicity probabilities, exemplifying a well-balanced efficacy–safety profile under the composite ranking scheme. Collectively, these findings demonstrate that SM-GAT enables rational early-stage lead identification through explicit multi-objective optimization. By jointly modeling multi-target efficacy and toxicity liabilities rather than optimizing a single endpoint, the framework provides a practical framework for prioritizing balanced candidates, which is particularly critical for long-term therapeutic areas such as type 2 diabetes mellitus.

## 4 Conclusion

This study presents SM-GAT, a safety-aware multi-task graph attention framework for multi-objective anti-diabetic lead discovery from Traditional Chinese Medicine. By jointly modeling dual-target efficacy (DPP4 and α-glucosidase inhibition) alongside acute oral and clinical toxicity endpoints, the framework directly addresses a central bottleneck in early drug discovery: the simultaneous optimization of therapeutic potency and safety liability. Systematic benchmarking demonstrates that multi-task learning substantially enhances predictive performance, particularly for data-scarce endpoints such as α-glucosidase inhibition. The observed gains suggest that shared representations spanning efficacy and toxicity domains may facilitate beneficial cross-task regularization while mitigating overfitting to endpoint-specific noise. Importantly, performance stability across activity thresholds and independent training runs suggests the robustness of the learned representations under heterogeneous and partially labeled conditions.

Beyond predictive accuracy, SM-GAT provides chemically interpretable attention patterns through atom-level attention analysis, highlighting substructures potentially associated with efficacy and toxicity predictions. This interpretability strengthens confidence in deployment for rational lead prioritization rather than purely black-box screening. Large-scale virtual screening of the TCMBank library further demonstrates practical utility. The identification of structurally diverse candidates with balanced efficacy–toxicity profiles, together with concordance between model predictions and traditional anti-diabetic knowledge, supports the applicability of SM-GAT for integrating traditional botanical resources with modern molecular informatics.

This work provides a safety-aware computational framework for natural product–driven multi-target drug discovery. By embedding toxicity awareness directly into early prioritization, SM-GAT may facilitate earlier identification of candidates with balanced efficacy and safety profiles in chronic metabolic disease therapeutics. SM-GAT is not proposed as a replacement for endpoint-specific specialized tools, but rather as a unified framework for simultaneous multi-endpoint screening. The advantage of jointly modeling efficacy and toxicity is demonstrated by improvements across several endpoints, particularly data-scarce tasks, supporting the value of a shared representation across heterogeneous endpoints. Future work will extend the framework to broader metabolic networks with experimental validation, while incorporating scaffold-based and temporal validation protocols to more rigorously assess chemical-space generalizability and address class imbalance *via* stratified batching or transfer learning.

## Data Availability

The original contributions presented in the study are included in the article/Supplementary Material, further inquiries can be directed to the corresponding author.
