# Supplementary material for: SM-GAT: a safety-aware multi-task graph attention network for multi-target anti-diabetic lead discovery from natural products
Source: Front Mol Biosci. 2026 Jul 8;13:1854162. doi: 10.3389/fmolb.2026.1854162 (PMC13388125; doi:10.3389/fmolb.2026.1854162)
Supplement: Supplementary file 1 [file DataSheet3.docx]

**SM-GAT: A Safety-Aware Multi-Task Graph Attention Network for Multi-Target Anti-Diabetic Lead Discovery from Natural Products**

Jianxin Zhang ^a^^[[1]](#footnote-1)^, Hongyi Liu ^b1^ and Shengnan Guo ^a^*

1. He Second Qilu Hospital of Shandong University, Shandong 250033, China.
2. The First Clinical School of Medicine, Yunnan University of Traditional Chinese Medicine, Yunnan 650500, China


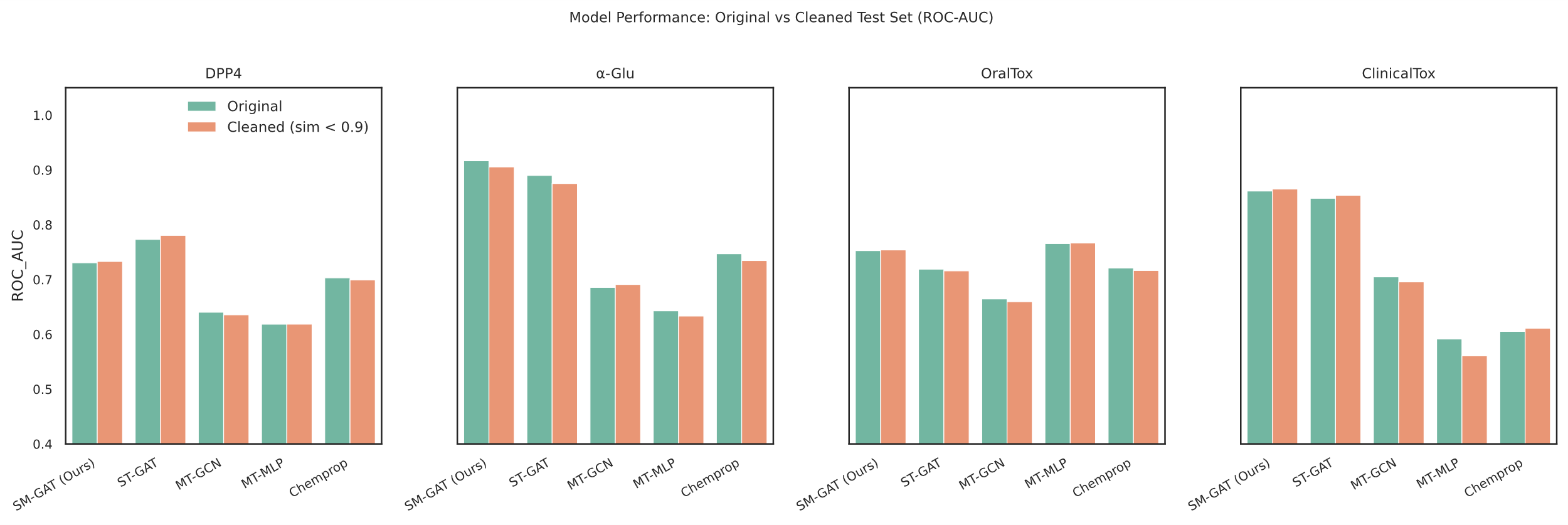


**Figure S1.** Performance comparison on the original test set and the cleaned test set. Note: The cleaned test set excludes compounds whose maximum Tanimoto similarity to the training or validation set exceeds 0.9.

**Table S1. Summary of prediction tasks, label distributions, and atom-level input features used in the SM-GAT framework.**

(A) Task-specific dataset statistics at the 500 nM bioactivity threshold. Positive and negative sample counts are reported for each endpoint. The total number of unique molecules reflects the union of partially overlapping task-specific datasets.

(B) Atom-level features derived from RDKit, including data types and chemical descriptions. Feature encoding was intentionally minimal to reduce descriptor bias and promote topology-driven representation learning.

(C) Distribution of compounds across training, validation, and test splits for each task. Positive, negative, and missing sample counts are reported for each task across training, validation, and test splits. Missing labels were masked during loss computation to accommodate heterogeneous label availability across tasks.

| (A) Task statistics and label distribution | | | | | | | |
| --- | --- | --- | --- | --- | --- | --- | --- |
| Task | | Total Samples | | Positive (1) | | Negative (0) | |
| DPP4 (500 nM) | | 4,887 | | 3,281 | | 1,606 | |
| alpha_glu (500 nM) | | 630 | | 80 | | 550 | |
| OralTox (LD_50)_ | | 9,843 | | 2,547 | | 7,296 | |
| ClinicalTox | | 1,406 | | 91 | | 1,315 | |
| Total unique molecules | | 16,669 | | | | | |
| (B) Atom-level features | | | | | | | |
| Feature Name | | Type | | Description  Normalized as $Z/10$ (e.g., C=0.6, N=0.7).  Number of neighboring atoms in the molecular graph.  Assigned formal charge based on RDKit analysis.  RDKit states (SP, SP2, SP3, SP3D, SP3D2) converted to integers.  1 if the atom is part of an aromatic system, 0 otherwise. | | | |
| Atomic Number | | Continuous | |  |  |  |  |
| Degree | | Discrete | |  |  |  |  |
| Formal Charge | | Discrete | |  |  |  |  |
| Hybridization | | Categorical | |  |  |  |  |
| Aromaticity | | Binary | |  |  |  |  |
| (C) Training/validation/test split details | | | | | | | |
| Task | Split | | Positive | | Negative | | Missing |
| DPP4 | Train | | 2,371 | | 1,160 | | 8,511 |
|  | Validation | | 418 | | 205 | | 1,503 |
|  | Test | | 492 | | 241 | | 1,768 |
| alpha_glu | Train | | 60 | | 399 | | 11,583 |
|  | Validation | | 8 | | 67 | | 2,051 |
|  | Test | | 12 | | 84 | | 2,405 |
| OralTox | Train | | 1,829 | | 5,282 | | 4,931 |
|  | Validation | | 314 | | 931 | | 881 |
|  | Test | | 404 | | 1,083 | | 1,014 |
| ClinicalTox | Train | | 64 | | 940 | | 11,038 |
|  | Validation | | 14 | | 184 | | 1,928 |
|  | Test | | 13 | | 191 | | 2,297 |

**Table S2. Optimized hyperparameter configurations of the SM-GAT model under different bioactivity thresholds.**

Grid search ranges and final selected hyperparameters are reported for activity thresholds of 100 nM, 500 nM, and 1000 nM. Model configurations were selected based on validation ROC-AUC performance while maintaining stability across tasks.

| Parameter | Search space | Final configuration | | |
| --- | --- | --- | --- | --- |
|  |  | 100 nM | 500 nM | 1000 nM |
| Hidden dimension | 64, 128 | 64 | 128 | 128 |
| Learning rate | 0.001, 0.0005 | 0.001 | 0.001 | 0.001 |
| Attention heads | 4, 8 | 8 | 8 | 8 |
| Dropout rate | 0.1, 0.2 | 0.2 | 0.2 | 0.2 |

**Table S3. Multi-metric performance evaluation of the SM-GAT model across different bioactivity thresholds.**

| Task / Threshold | 100 nM (Mean ± Std) | 500 nM (Mean ± Std) | 1000 nM (Mean ± Std) |
| --- | --- | --- | --- |
| DPP4 | 0.729 ± 0.015 | 0.767 ± 0.009 | 0.744 ± 0.022 |
| alpha_glu | 0.914 ± 0.064 | 0.894 ± 0.030 | 0.865 ± 0.051 |
| OralTox | 0.693 ± 0.030 | 0.708 ± 0.013 | 0.707 ± 0.017 |
| ClinicalTox | 0.853 ± 0.044 | 0.848 ± 0.038 | 0.828 ± 0.043 |

**Table S4. Detailed Performance Metrics for the Optimal Model (500 nM Threshold).**

|  | DPP4 | alpha_glu | OralTox | ClinicalTox_label |
| --- | --- | --- | --- | --- |
| Seed 1 | 0.762893 | 0.910256 | 0.707901 | 0.824675 |
| Seed 2 | 0.758918 | 0.852137 | 0.692517 | 0.901736 |
| Seed 3 | 0.765048 | 0.924031 | 0.713963 | 0.847542 |
| Seed 4 | 0.765672 | 0.8722 | 0.726995 | 0.86246 |
| Seed 5 | 0.782904 | 0.910256 | 0.701007 | 0.801388 |

**Table S5. Performance comparison between SM-GAT and single-task GAT models.**

Mean ± standard deviation over five independent runs (500 nM threshold).

| Task | Metric | SM-GAT | Single-task GAT |
| --- | --- | --- | --- |
| alpha_glu | ROC-AUC | 0.892 ± 0.057 | 0.878 ± 0.071 |
| alpha_glu | PR-AUC | 0.684 ± 0.096 | 0.654 ± 0.147 |
| DPP4 | ROC-AUC | 0.751 ± 0.021 | **0.766 ± 0.005** |
| DPP4 | PR-AUC | 0.844 ± 0.013 | **0.846 ± 0.010** |
| OralTox | ROC-AUC | 0.744 ± 0.010 | 0.718 ± 0.017 |
| OralTox | PR-AUC | 0.515 ± 0.025 | 0.497 ± 0.037 |
| ClinicalTox | ROC-AUC | 0.849 ± 0.020 | 0.849 ± 0.007 |
| ClinicalTox | PR-AUC | 0.239 ± 0.070 | 0.213 ± 0.026 |

1. * Corresponding author: [tinaoma@163.com](mailto:tinaoma@163.com) (S. Guo).

   These authors contributed equally to this work. [↑](#footnote-ref-1)
